# Sea Cucumber Detection Algorithm Based on Deep Learning

**DOI:** 10.3390/s22155717

**Published:** 2022-07-30

**Authors:** Lan Zhang, Bowen Xing, Wugui Wang, Jingxiang Xu

**Affiliations:** 1 College of Engineering Science and Technology, Shanghai Ocean University, Shanghai 201306, China; zhang258708921@163.com (L.Z.); jxxu@shou.edu.cn (J.X.); 2 Shanghai Investigation Design & Research Institute, Shanghai 200335, China; 3 China Ship Development and Design Center, Wuhan 430064, China; woxinyouyou99@163.com

**Keywords:** sea cucumber fishing, image recognition, deep learning, single-shot multibox detector

## Abstract

The traditional single-shot multiBox detector (SSD) for the recognition process in sea cucumbers has problems, such as an insufficient expression of features, heavy computation, and difficulty in application to embedded platforms. To solve these problems, we proposed an improved algorithm for sea cucumber detection based on the traditional SSD algorithm. MobileNetv1 is selected as the backbone of the SSD algorithm. We increase the feature receptive field by receptive field block (RFB) to increase feature details and location information of small targets. Combined with the attention mechanism, features at different depths are strengthened and irrelevant features are suppressed. The experimental results show that the improved algorithm has better performance than the traditional SSD algorithm. The average precision of the improved algorithm is increased by 5.1%. The improved algorithm is also more robust. Compared with YOLOv4 and the Faster R-CNN algorithm, the performance of this algorithm on the P-R curve is better, indicating that the performance of this algorithm is better. Thus, the improved algorithm can stably detect sea cucumbers in real time and provide reliable feedback information.

## 1. Introduction

Recently, sea cucumber farming has been rapidly developed as an aquatic type of farming [1]. With the development of sea cucumber production, the breeding problems of sea cucumbers are becoming increasingly serious. Traditional sea cucumber fishing, which has low efficiency and high risk, is mainly dependent on manual work [2]. To promote the development of sea cucumber breeding automation, it is necessary to research the automatic identification of sea cucumbers in the natural underwater environment based on machine vision [3,4]. A sea cucumber target recognition by BP neural networks was proposed by Wang et al. They used RGB and depth images as prior knowledge to improve recognition accuracy [5]. A depth residual network with different configurations for sea cucumber target recognition was proposed by Guo et al. [6]. A real-time cultured sea cucumber detector attached to an autonomous underwater vehicle (AUV), with YOLOv4-tiny and transfer learning are proposed by Thao et al. [7]. After the study of sea cucumber target recognition, the above scholars and other researchers have proposed a series of methods with practical applications. However, the majority of research has not considered the application to embedded platforms and real-time issues.

To solve the above problems, an improved SSD target detection algorithm is proposed. First, we use the MobileNetv1 to detect and locate sea cucumbers. Secondly, the shallow feature receptive fields are improved by RFB, and have more details and location information of small targets. This algorithm combines the attention mechanism to strengthen features at different depths, suppress irrelevant features, and perform feature fusion to further improve the accuracy of sea cucumber detection.

## 2. SSD Object Detection Algorithm

The core of the traditional SSD algorithm is to predict results by different convolution layers. The traditional SSD algorithm introduces the idea of regression and proposes the concept of the prior box [8]. The algorithm predicts targets on feature maps of different receptive fields, and completes target location and classification at one time.

### 2.1. Traditional SSD Model Structure

**Figure 1:**
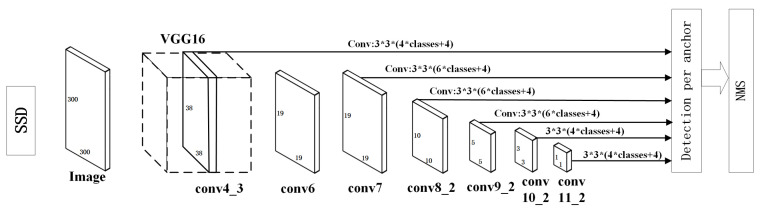
Network-structure diagram of traditional SSD algorithm.

A traditional SSD network uses VGG16 as the backbone network. The two fully connected layers of VGG16, which lay the foundation for subsequent multi-scale feature extraction, are converted into convolution layers. The traditional SSD network has six feature maps at different dimensions. Conv4_3, Conv7, Conv8_2, Conv9_2, Conv10_2, and Conv11_2 are connected to the final classification layer for regression prediction [9,10]. The network structure of the traditional SSD algorithm is shown in Figure 1.

First, the traditional SSD algorithm generates six prior boxes on six feature maps. Secondly, the prior boxes are assembled from different feature maps. Finally, the final set of prior boxes is selected by non-maximum suppression (NMS) [11,12].

### 2.2. The Loss Function of Traditional SSD

Model prediction performance is tested by the loss function [13,14]. The traditional SSD algorithm loss function is divided into two parts, the classification loss function and the position loss function,

(1) $$L\left(x,c,l,g\right)=\frac{1}{N}\left(L_{conf}\left(x,c\right)+\alpha L_{loc}\left(x,l,g\right)\right),$$

where $L_{conf}$ is the classification loss function, $L_{loc}$ is the position loss function, *N* is the number of samples, α is the weighting coefficient, *x* is the matching information for the current prediction box category, *c* is the labeled category, *l* represents the coordinates of the search prediction boxes, and *g* represents the coordinates of marked boundary frames.

The position loss function is shown as follows. Where $x_{ij}^{k}=\left\{1,0\right\}$ represents whether the *i* search prediction box matches the *J* real box on category *k*, $l_{i}^{m}$ is the prediction box; ${\hat{g}}_{j}^{m}$ is the ground-truth box, *N* is the number of matched samples, Pos is the positive sample, Box is a set of prediction box attribute parameters, and $smooth_{L1}$ is the error function of L1:

(2) $$L_{loc}\left(x,l,g\right)=\sum_{i\in Pos}^{N}\sum_{m\in Box}x_{ij}^{k}smooth_{L1}\left(l_{i}^{m}-{\hat{g}}_{j}^{m}\right).$$

The classification loss function is shown as follows. Where ${\hat{c}}_{p}^{i}$ is the probability which is the target of the *i* prediction box is *p*, ${\hat{c}}_{0}^{i}$ is the probability that the target is not detected in the *i* prediction frame, $x_{ij}^{p}$ represents whether the *i* search prediction box matches the *j* real box on category *P*, Neg is the negative sample, and Pos is the positive sample:

(3) $$L_{conf}\left(x,c\right)=-\sum_{i\in Pos}^{N}x_{ij}^{p}\log\left({\hat{c}}_{i}^{p}\right)-\sum_{i\in Neg}\log\left({\hat{c}}_{i}^{0}\right)$$

(4) $${\hat{c}}_{i}^{p}=\exp\left(c_{i}^{p}\right)/\sum_{p}\exp\left(c_{i}^{p}\right).$$

### 2.3. Traditional SSD Performance Analysis

The traditional SSD algorithm uses multi-scale feature maps for detection, and generates six prior boxes which have different aspect ratios [15].

**Figure 2:**
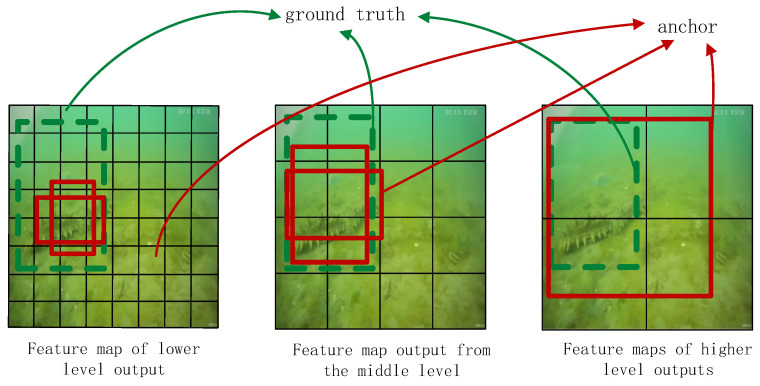
Output results from different levels of SSD.

As shown in Figure 2, feature maps of the lower level are larger, but the receptive field of each unit is relatively small. Feature maps of the lower level are suitable for detecting small targets. Feature maps of the higher level are smaller, but the receptive field of each unit is relatively large. Feature maps of the larger level are suitable for detecting large targets.Therefore, the detection of small targets relies on lower-level feature maps. The number of lower-level-feature convolution layers is small, resulting in insufficient feature extraction and poor semantic distinction.

**Figure 3:**
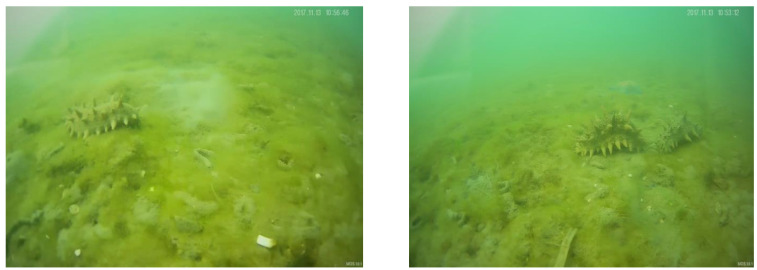
Underwater robots taking photographs of sea cucumbers.

As shown in Figure 3, the sea cucumber is a small target. When using the traditional SSD algorithm to identify sea cucumbers, the detection ability is insufficient, the robustness is poor, and it is impossible to accurately locate the underwater sea cucumber. In addition, the traditional SSD algorithm has a large computing capacity, and cannot be applied to embedded platforms. To overcome these problems, the traditional SSD algorithm is optimized.

## 3. Sea Cucumber Detection Algorithm Based on Improved MobileNetv1 SSD

### 3.1. MobileNetv1 Structure

MobileNetv1, which uses depthwise separable convolution, is a lightweight CNN structure [16]. The depthwise separable convolution is mainly divided into two parts, depthwise convolution and pointwise convolution [17,18].

**Figure 4:**
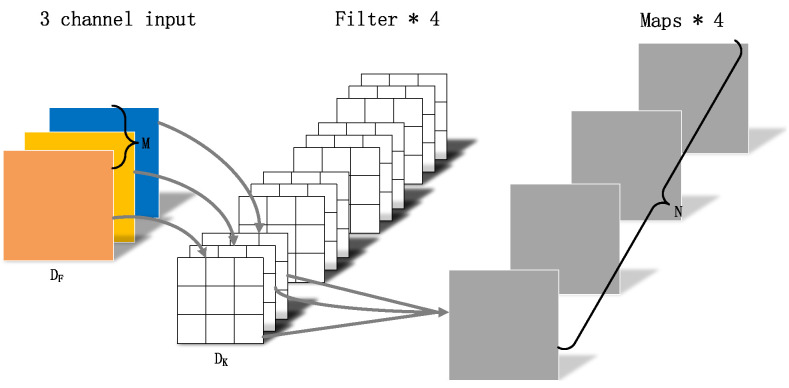
Standard convolution.

**Figure 5:**
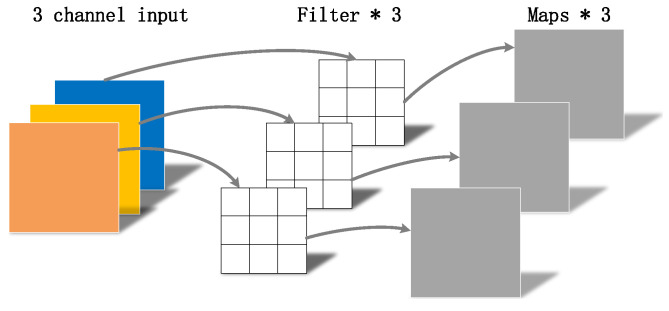
Depthwise convolution.

**Figure 6:**
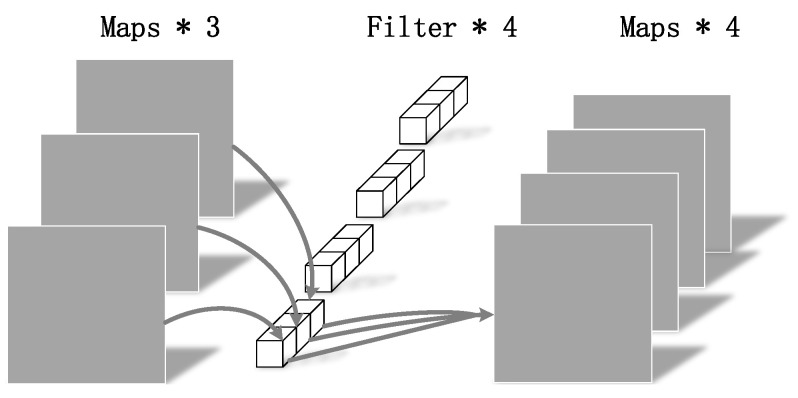
Pointwise convolution.

As shown in Figure 4, Figure 5 and Figure 6,where $D_{F}$ represents the height and width of the input matrix, $D_{K}$ represents the size of the convolution kernel, *M* is the number of input feature matrix channels, and *N* is the number of output feature matrix channels:

(5) $$\frac{D_{K}\times D_{K}\times D_{F}\times D_{F}\times M+M\times N\times D_{F}\times D_{F}}{D_{K}\times D_{K}\times M\times N\times D_{F}\times D_{F}}=\frac{1}{N}+\frac{1}{D_{K}^{2}}.$$

The depthwise separable convolution in MobileNetv1 networks uses a convolution kernel of 3 × 3. The computation of standard convolution parameters is about nine times that of depthwise separable convolution parameters. The introduction of the MobileNetv1 network reduces the number of calculation parameters and realizes a lightweight structure.

### 3.2. Introduction of SSD Network with Dilated Convolutional Structure

The RFB module, which is similar to the inception network, is a multi-branch convolution block [19]. The RFB module consists of a multi-branch convolution layer and a dilated convolution layer. The dilated convolution adds holes to increase the receptive field. The dilated convolution has a hyper-parameter $K_{d}$ [20,21]:

(6) $$K_{d}=l\times \left(K-1\right)+1,$$

where $K_{d}$ is the size of the dilated convolution kernel, *L* is a dilation factor, and *K* is the size of the original convolution kernel.

**Figure 7:**
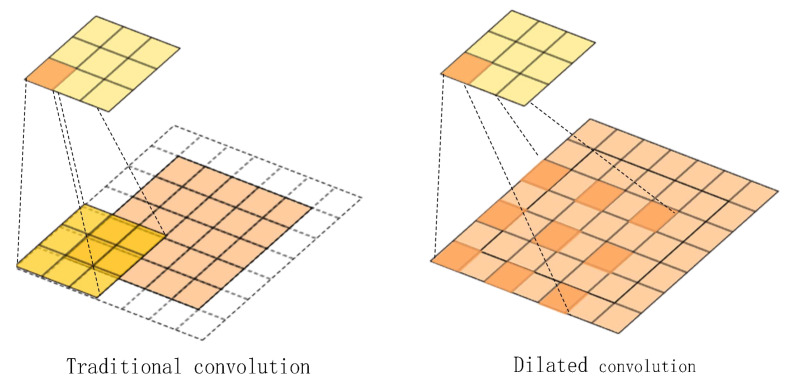
Comparison of traditional and expansive convolution.

As shown in Figure 7, when the dilation factor is 2, the dilated convolution has larger receptive fields than the original convolution.

**Figure 8:**
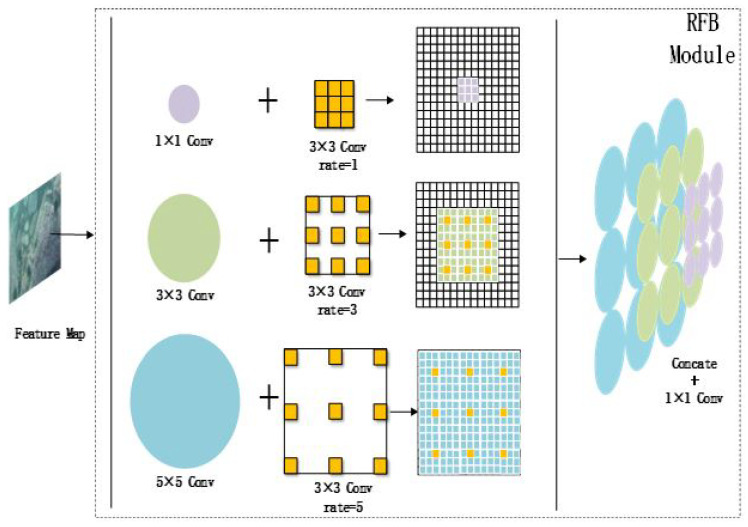
RFB module processing flowchart.

The RFB module processing flow is shown in Figure 8. The RFB module consists of three branch modules [22]. The bottom layer in each branch is processed by convolutional nuclei of 1 × 1, 3 × 3, and 5 × 5. Finally, three different receptive fields are obtained [23,24].

In this paper, the RFB module is introduced to process the Conv4_3 layer and the FC7 layer. As a result, shallow semantic information is richer. Deep feature maps need feature processing, so introducing an attention mechanism to strengthen the feature maps at different depths.

### 3.3. Introduction of Attention Mechanisms

In this paper, spatial attention and channel attention mechanisms are introduced to strengthen the features at different depths [25]. Finally, the multi-layer feature maps are fused to achieve better detection results.

First, the spatial attention mechanism is introduced into the features at different depths. Secondly, when a multi-channel feature map is an input, spatial attention will learn and train relationships of different spatial domains in the feature map. After giving higher weights to more representative local features, a two-dimensional spatial weight map *W* is generated. Finally, the two-dimensional weight map *W* is multiplied by the corresponding position space to obtain a representative feature map. The training mechanism of spatial attention is as follows. First, through global maximum pooling and global average pooling, feature representative values are obtained at each spatial position. Then, these feature representative values are fused by convolution operation to obtain the spatial attention map. Finally, spatial attention weights of 0–1 are generated by the sigmoid activation function [26,27,28]. The calculation formula is as follows:

(7) $$W_{s}\left(F\right)=sigmoid\left\{f^{3\times 3}\left\{\left[MaxPool\left(F\right);AvgPool\left(F\right)\right]\right\}\right\},$$

where $W_{s}\left(F\right)$ is the feature map after spatial attention mechanism processing, *F* is the input multi-channel feature map, $f^{3\times 3}$ is the convolution operation of 3 × 3, AvgPool is the global average pooling, and MaxPool is the global maximum pooling.

First, the channel attention method filters out irrelevant channel features in a multi-channel feature map. Secondly, through the relationship between each channel in the feature map to learn the weight array. Finally, the weight array is multiplied by the corresponding channel [29,30]. The following formula is used to calculate the channel attention mechanism:

(8) $$W_{s}^{\prime }\left(F\right)=sigmoid\left\{MLP\left[AvgPool\left(F\right)\right]+MLP\left[MaxPool\left(F\right)\right]\right\},$$

where $W_{s}^{\prime }\left(F\right)$ is the result characteristic diagram, MLP is the multilayer perceptron, AvgPool is the global average pooling, and MaxPool is the global maximum pooling.

### 3.4. MobileNetv1 SSD Network with Attention Mechanisms

The improved SSD algorithm can detect sea cucumbers by the MobileNetv1 SSD network. The size of the shallow feature receptive fields is increased by RFB [31]. First, the attention mechanism creates a model of the relationship between relevant feature channels and feature spaces. Secondly, the obtained weights between each feature channel and feature space, are multiplied by the original feature information. Finally, the obtained channel features map and spatial feature map not only contain the most representative features but also suppress irrelevant features. In short, the improved SSD algorithm not only improves the recognition accuracy of small target objects but also reduces the missed detection rate and false detection rate.

**Figure 9:**
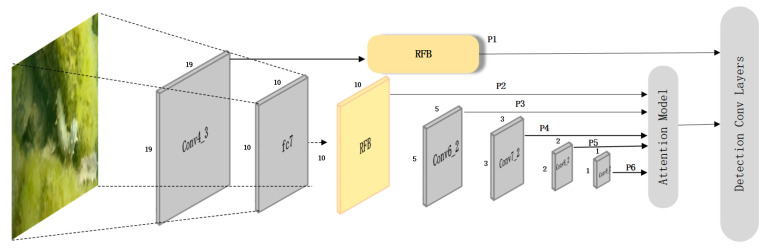
Improved SSD network structure.

In this paper, the features of the Conv4_3 and FC7 layers are selected to utilize RFB. Finally, P1(19 × 19), P2(10 × 10), P3(5 × 5), P4(3 × 3), P5(2 × 2), and P6(1 × 1) feature maps are obtained [32]. The improved SSD network structure is shown in Figure 9.

## 4. Experimental Results and Analysis

To verify the feasibility of the proposed algorithm, the following computing environment was used: Intel(R)Core(TM)i7-9750H CPU, NVIDIA GeForce RTX 2060 graphical processing unit, Ubuntu 20.04 operating system, and Keras 2.1.5 deep-learning framework.

### 4.1. Experimental Data

The experimental data were extracted from a video of shallow sea cucumber farming. The video is recorded by a remotely operated vehicle. By framing the video, 1710 original sea cucumber images were collected by using data augmentation to increase the original dataset. The number of images in the dataset is 4005. The filtered images were manually annotated by LabelImg software. In the dataset, 70% comprise the training set, and 30% comprise the test set. LabelImg software uses rectangular boxes to mark sea cucumber targets. The label information is stored in XML format. The information includes image name, category name, image size, and position information.

### 4.2. Evaluation Index Setting

**Figure 10:**
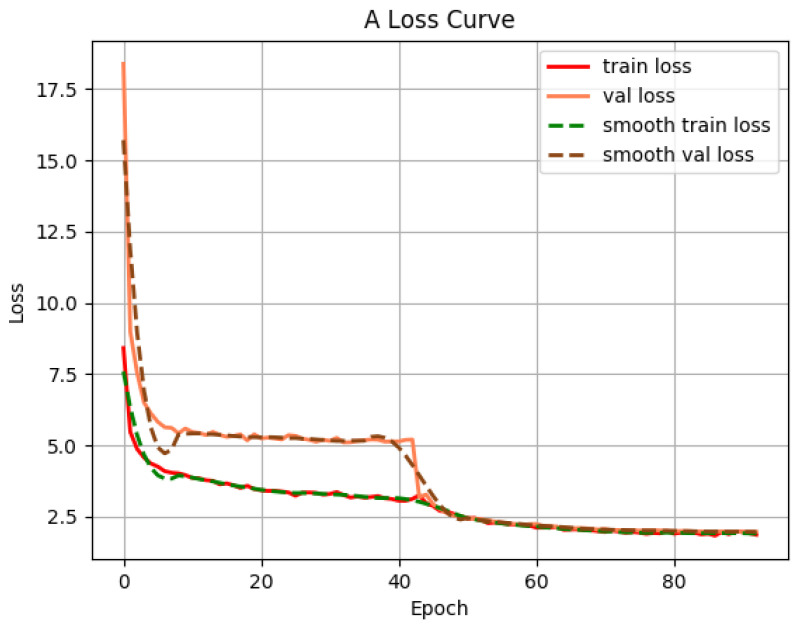
The loss curve.

The intersection over union, which is the overlap rate between the candidate bound and the ground truth bound, is a concept in object detection. Owing to the complexity of the marine environment, this paper uses mean average precision (mAP) with an IOU threshold of 0.5 and detection frame rate as evaluation indicators. The mean average precision, which is calculated by the P-R curve, is an evaluation metric in object detection models. The P-R curve consists of a precision curve and a recall curve. The P-R curve reflects global performance [33]. The following equations are used to calculate precision, recall, and mAP:

(9) Precision=TP/(TP+FP)

(10) Recall=TP/(TP+FN)

(11) $$mAP=\sum_{i=1}^{k}AP_{i}/k,$$

where FP is a false positive example, FN is a false negative example, TP is the real example, TN is a true negative example, $AP_{i}$ is the average precision of a category; and *k* is the number of categories. Figure 10 is a loss curve.

### 4.3. Improved SSD Model Validation

To verify the effectiveness of the improved SSD model, this paper conducts a comparative experiment on the recognition effect of the traditional SSD model. The improved SSD model uses the same training set and sets the same parameters as the traditional SSD model. The training is divided into the freezing stage and the thawing stage. In the freezing stage, the learning rate is $5\times 10^{4}$, the model backbone is frozen, and the network is fine-tuned. In the thawing stage, the learning rate was $1\times 10^{4}$, the model backbone is thawed, and the feature extraction network is adjusted.

As shown in Table 1, compared with the traditional algorithm, the average accuracy of the algorithm proposed in this paper is improved by 5.1%, the detection frame rate is improved by 18.514 frame/s. The detection accuracy of sea cucumber is improved effectively.

**Figure 11:**
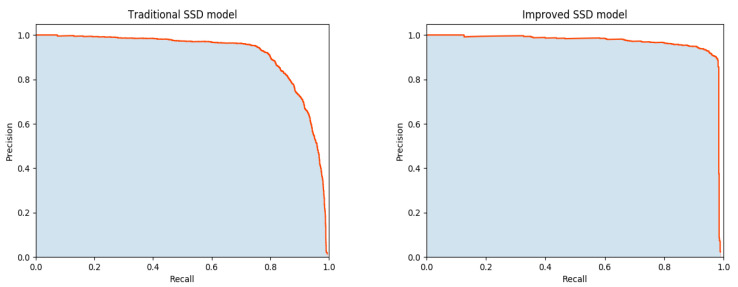
Comparison of P-R curves before and after model improvement.

Figure 11 is the comparison chart of the P-R curve before and after the model improvement. The model performance is reflected by the P-R curve.

As shown in Figure 11, the improved SSD model has the larger area between the P-R curve and coordinate axis. The equilibrium point of the improved SSD model is closer to coordinate (1,1), indicating that the system performance of the improved SSD model is better.

**Figure 12:**
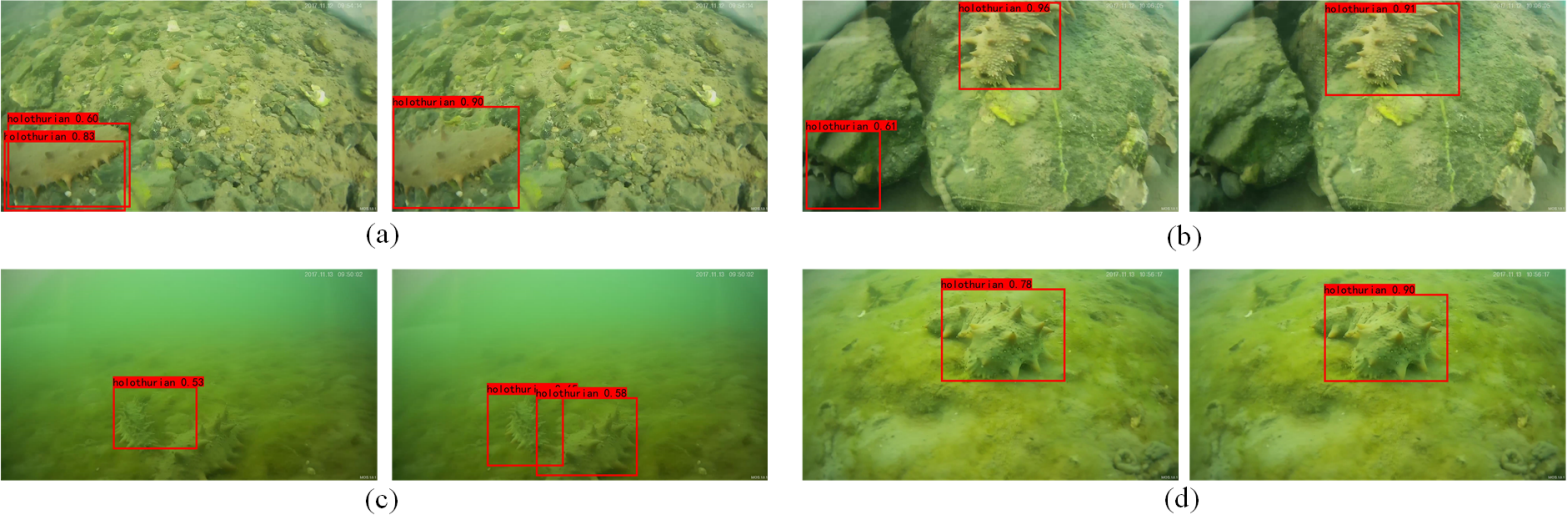
Comparison of sea cucumber identification before and after model improvement.

Figure 12 shows the comparison images of sea cucumbers detected by the SSD model before and after improvement. As shown in Figure 12a, the result of the traditional SSD algorithm has a repeat box. As shown in Figure 12b, the result of the traditional SSD algorithm has a false detection. As shown in Figure 12c, the result of the traditional SSD algorithm reveals a missed detection. The comparison results in Figure 12d show that the confidence of the improved SSD algorithm has increased by 12%. According to the results in Figure 12, the proposed algorithm reduces the missed detection rate. Compared with the traditional algorithm, the confidence of the proposed algorithm is higher. In addition, the portion of the target missed by the traditional SSD algorithm can be detected by the proposed algorithm.

### 4.4. Comparison of Different Models

**Figure 13:**
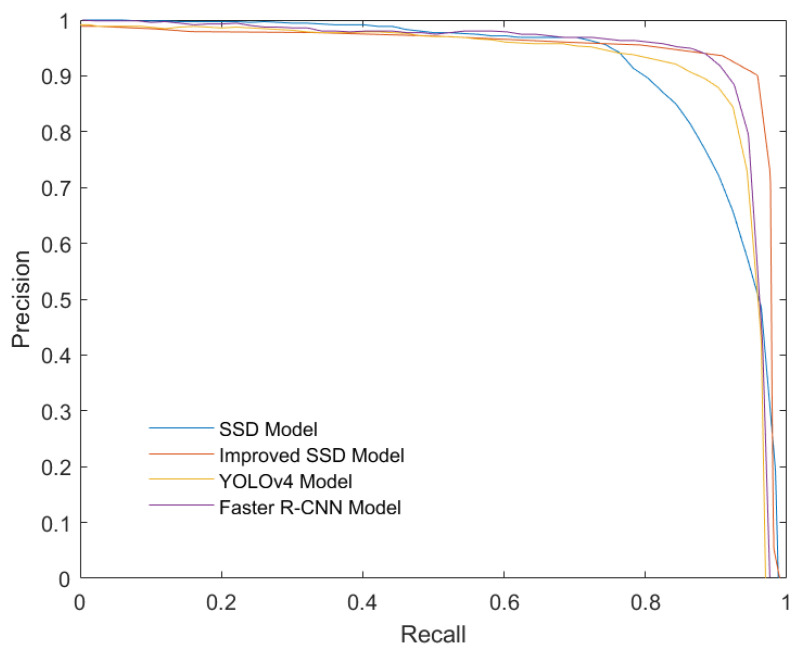
Four models comparison of P-R curves.

In order to further prove the effectiveness of the algorithm in this paper, the FasterRCNN algorithm and the YOLOv4 algorithm are selected for comparison test. The Faster RCNN algorithm and the YOLOv4 are typical one-stage and two-stage target detection algorithms. The following graph is the P-R curve comparison graph of four algorithms. Under the same sea cucumber dataset, the performance of the proposed algorithm on the P-R curve is better than YOLOv4 and Faster R-CNN, indicating that the performance of the proposed algorithm is better (see Figure 13). In terms of detection speed, in the process of algorithm testing, the proposed algorithm is slower than YOLOv4 algorithm and faster than FasterR-CNN algorithm. Compared with the typical YOLOv4 and FasterR-CNN in the first and second stages, the proposed algorithm has better target detection ability and higher applicability for sea cucumber target recognition.

## 5. Conclusions

Aimed at the low accuracy and a large amount of computation required by traditional SSD algorithms in detecting sea cucumbers, an improved algorithm for sea cucumber detection is proposed. First, a MobileNetv1 SSD network is used to detect and locate sea cucumbers. Through a receptive field block, the shallow feature receptive field is improved to increase the detail and location information. The improved algorithm is combined with an attention mechanism to strengthen features of different depths. The experimental results show that, compared with the traditional SSD algorithm, the proposed algorithm has good robustness and recognition rate. Compared with the YOLOv4 and Faster R-CNN algorithms, the performance of this algorithm on the P-R curve is better, indicating that the performance of this algorithm is better. Underwater sea cucumbers have the characteristic of changing body color to match their environment, and future research will aim at solving the problems caused by this characteristic, focusing on finding an innovative pre-treatment method to achieve efficient identification of sea cucumbers.

## Figures and Tables

**Table 1:** Improved and traditional SSD algorithm comparison test results.

Detection method	${\mathrm{mAP}}_{50}$	Frames per Second	No. of Iterations
Traditional SSD algorithms	0.914	5.916	100
Improved algorithm	0.965	24.430	100

## Abbreviations

The following abbreviations are used in this manuscript:

SSD	Single Shot MultiBox Detector
RFB	Receptive Field Block
